# Selections and Abstracts

**Published:** 1914-12

**Authors:** 


### SELECTIONS AND ABSTRACTS

### NEOPLASMS OF THE BLADDER.*

### WITH REMARKS ON OPERATIVE TREATMENT.

By Francis R. Hagner, M. D., F. A. C. S., Washington.

It might be well to say, first, a few words concerning the so-called non-malignant neoplasms of the bladder Papilloma is the most common type of benign tumor; the other less frequent forms are fibroma, myoma and cysts. The papillomata that show no tendency to infiltrate the bladder wall; and microscopically show no evidence of malignancy, are considered by some authorities to be benign growths.

I am sure, however, that we should combat this idea and so make fewer mistakes than if we consider them benign. A large proportion of these tumors do infiltrate the bladder wall, and lead to metastasis. Some that are apparently benign in the beginning tend later to infiltrate the bladder wall; some that do not show any tendency to infiltrate, tend to multiply and destroy life by hemorrhage, sepsis, and interference with the renal function. I feel that if the term, benign tumors of the bladder, was eradicated, it would be a great advantage, as it gives too many men unfamiliar with them the impression that there is a class of bladder tumors clinically benign, whereas all tumors of the bladder are clinically malignant. Unless they are removed by some operative means, they will either directly or indirectly cause the death of the patient. Cysts of the bladder, fibromata, and myomata are so rare as to require no more than mention. The operative treatment of the papilloma is divided into excision by open operation and intravesical operation. The open operations in multiple papillomata have not been very satisfactory, as metastases are so apt to occur along the whole line of incision, very similar to keloid seen in the negro. Nitze's results in the intravesical operation on these growths gave a smaller percentage of recurrences than those operated on by the open method. I believe this to be especially true in the multiple papillomata.

There are a few cases reported of suprapubic removal, the base cauterized by the Oudin current, that have given favorable results. Unquestionably the majority of genitourinary surgeons are using either excision with an operating cystoscope followed by cauterization, or else the high frequency cauterization by the Oudin current. We have had several of these cases, and while they have not extended over a period sufficient to make accurate conclusions, and while small recurrences have been noted in two of the patients, they have been readily removed by another application of the current. The work of Doctor Buerger, Doctor Beer and Doctor Keyes along this line is well known. No one is yet able to speak positively on the value of this treatment; but all agree at this time that it seems to offer better chances of success than any of our earlier treatments. It is imperative to examine these cases cystoscopically at regular intervals, and remove at once any evidence of recurrence.

The malignant tumors of the bladder are divided into sarcomata and carcinomata. Sarcomata is less common than carcinoma. In quite an experience with malignant disease of the bladder we have had only two cases of small round cell sarcoma. Authorities seem to agree that sarcomata are much more malignant than the carcinomata; one of our patients died four months after excision of one tumor including one-third of the bladder, from local and general metastasis, the local recurrence beginning before the patient left the hospital in the healing portion of the vesical wall. The second patient is alive, but has a recurrence.

The bladder is one of the most unfortunate locations for carcinoma, as it presents many difficulties both as regards radical and palliative measures of treatment. As to the frequency of malignant tumors of the bladder in relation to those in other parts of the body, some authors give 3.9 per cent and others 7.6 per cent, so there is some variance in personal experience by different observers. I have been fortunate or unfortunate in
There are two varieties, one where the growth is primary seeing a number of these distressing cases.
gans. We will consider only the primary form. Carcinomatous
and the other secondary through metastasis from adjacent or-
disease of the bladder may unquestionably last for some time without metastasis, but even this fact does not lessen the gravity of the situation and the only hope for a successful outcome is early diagnosis. It might be advisable briefly to mention the symptoms of vesical carcinoma. I purposely did not take up the symptoms of nonmalignant growths, as the symptoms of the malignant are identical.

Hematuria is the cardinal symptom, but is often not present until the case is far advanced, especially if the tumor is away from the vesical outlet. The hemorrhage has distinct characteristics, being generally abundant, of long duration, and occurring irrespective of injury, violent efforts, or straining, obstinate in yielding to remedies, and finally disappearing for as little apparent reason as it took place. It may recur in a few days or not recur for several months. As a general rule, carcinomatous tumors give more persistent bleeding than the so called nonmalignant types of bladder tumor. But in the papilloma the bleeding, when it occurs, is more profuse. The hemorrhage is terminal, resulting from bladder contraction.

Pain is a most inconstant symptom; it may be entirely absent, but later on it usually becomes severe and constant. Tumors distant from the urethral orifice produce less pain than those at the orifice. Reflex pains due to pressure on the nerves are very common in this condition, radiating to the thighs, anus, perineum and hypogastrium, but are usually a late symptom. The pain in the bladder is nearly always due to the accompanying cystitis and is always present sooner or later. The tumor mass may be so large as to act as a foreign body and cause pain.

Fragments of the tumor appear in the urine. If the growth is so situated as to interfere with the ureteral outlet, hydronephrosis will develop. Just such a case has recently been seen with Doctor Cuthbert. I had previously read two papers on this subject and described the operation that has been employed in these cases. I have had three additional cases since the last report, one involving the vertex and the others the left lateral wall and fundus. Although the vertex of the bladder is supposedly a comparatively rare site for new growths, I cannot help but feel from my personal experience that they occur more frequently than is usually described. Statistics show that the lower third of the bladder is the site of two-thirds of vesical tumors; if this is true, the operation that I have used in these cases is applicable in one-third of all malignant tumors of the bladder. One of two facts is certainly true; either bladder tumors are more frequent, or else our methods of diagnosis at this time are more accurate.

I have seen more cases of bladder tumor in the past three years than in an entire previous professional experience of eighteen years. One would think it very easy to diagnose a tumor of the fundus of the bladder on cystoscopic examination, but I feel sure, in one of my cases with a broad sessile growth, the condition would have been overlooked had I not been suspicious of its presence and hence searched with care for the neoplasm.

Surgical treatment in carcinoma is divided into palliative and radical.

Palliative Treatment—1. The Oudin current seems to cause tumors to grow less rapidly and does relieve hemorrhage.

2. Suprapubic cystotomy and removal of the growth without extirpating the bladder wall, followed by cauterization is unsatisfactory and the tumor always recurs. A few cures have been reported by this method where the operation has been followed by treatment with radium.

3. Suprapubic cystotomy is used for drainage only in inoperable cases, relieving pain and tenesmus, giving relief similar to artificial anus in cancer of the bowel.

4. Double nephrotomy is done with ligation of ureter or drainage in the flank by ureterostomy for relief of tenesmus accompanying urination.

Radical Surgical Treatment.—1. Total extirpation of the bladder.

2. Excision of the bladder wall containing tumor mass, when the base of the bladder is involved, best done by intraperitoneal operation. By this method much better exposure of the bladder base is obtained, it being usually necessary to remove the ureter of the affected side and transplant it to a healthy portion of the bladder.

3. Cases that I am especially interested in are those involving the fundus or lateral wall of the bladder.

All my cases except the first have been operated in as described in the Annals of Surgery for November 1910. My inability to remove the friable mass in Case 1, without breaking the tumor mass, suggested to me the method of removal that was resorted to in the subsequent cases. In our first case, the patient had a very large flabby bladder, and although the bladder wall was retracted as soon as the incision was made, the wall collapsed, and it was found to be impossible to determine the site for the incision around tumor mass without inserting the finger in the viscus; although this was done as carefully as possible the tumor, being very friable, began to break up. I believe it is usually conceded that one of the disadvantages which attend the methods generally employed for removal of new growths for this viscus, is the production of trauma to the tumor mass during its removal. It is possible, however, with proper care to remove tumors from the fundus and lateral walls of the bladder without the slightest trauma to the tumor mass by the method used in these cases.

The operation is impracticable for tumors of the bladder base. The following is a description of the operation employed: The bladder is irrigated, and if bleeding, adrenaline, one to 10,000, is instilled and allowed to remain five minutes. The bladder is then distended with salt solution, and a Nitze cystoscope is introduced. This is held in place by an assistant while a suprapubic incision down to the bladder wall is made. The prevesical fat is well separated and the tissues are well drawn back by wide lateral retractors so as to give a good exposure of the bladder wall. I thought at this stage it might be possible by illuminating the bladder with the cystoscope to outline the tumor mass by the transmitted light; although the iridescence was caused by the illumination of the fluid, the outline of the tumor mass could be made out. The growth is then inspected through the cystoscope, the left hand holding the cystoscope; with the right a threaded needle is pressed on the fundus of the bladder, and the dimpling caused thereby is readily seen through the cystoscope. The needle is carried first to the left of the growth at a sufficient distance to give a margin of healthy bladder wall. It is then plunged into the bladder and the suture drawn through, being held taut by an assistant. The same procedure is employed at the right of the growth and at the lower border.

An incision is made through the bladder wall around the inner side of the three traction sutures, the portion of the bladder wall to be removed being clamped as the incision is advanced. The bladder wall containing the growth is lifted up by the clamp and held by an assistant. The fluid left in the bladder is removed by a large syringe through the suprapubic wound to prevent its entrance into the peritoneal cavity, and the cavity of the bladder is packed with gauze.

If the parietal peritoneum is to be removed (and I feel sure, if it is adherent to the bladder wall, that it should be), the incision in the bladder wall is then carried upward into the peritoneal cavity and the portion of the peritoneum covering the growth, is removed. The bladder and peritoneal wounds are then closed by two rows of chronic gut sutures, a suprapubic drain being left in the bladder, although in my last case the bladder was completely closed and drained by a retention catheter through the urethra. If the lateral wall is the seat of the growth, it may not be necessary to go into the peritoneal cavity; as in my case with the growth in this place, the peritoneum was easily stripped off the bladder wall, it is safer to carry the incision upward directly into the peritoneal cavity and remove the peritoneum covering the bladder over the site of the growth. It is very easy to do this, as the loose folds of the parietal peritoneum in this locality allow of easy approximation. From my experience in my last case, it would seem perfectly safe to close the bladder wound completely, if the patient can stand the retention catheter. For report of previous cases, see Annals of Surgery, November 1912.

Case V. Capt. D., aged seventy-seven years, operated on twenty years ago for stricture and extravasation of urine. January 25, 1912, I operated for a recurrent stricture involving the urethra from meatus to cut off muscle, through which only a filiform bougie could be passed. He left the hospital, February 19, 1912, in good condition, although he had five ounces of residual urine and definite prostatic enlargement. His history suggested that the urine was purulent for many years. After the operation the purulent condition of his urine continued, but in a less degree, and the patient voided his urine every four hours. He had had no previous history of bleeding, but, March 14th, forty-four days after operation for stricture, he presented himself reporting blood in his urine; his bladder was irrigated and the fluid returned stained with blood. Two days later he was cystoscoped at Garfield Hospital and a growth, five cm. in diameter, was noted at the junction of the fundus and the left lateral wall. A diagnosis of carcinoma was made. As the patient had marked arteriosclerosis, a phthalein test was made and we obtained thirty-five per cent in one hour. He was operated upon March 18, 1912. With nitrous oxide and oxygen anesthesia and infiltration of one to 400 novocaine into skin and subcutaneous tissue, the operation was performed as previously described. As the growth extended into the lateral wall of the bladder, but two suture points could be obtained by viewing with the cystoscope, the lateral wall being well freed from the surrounding tissue before the incision was made. This operation differed from the others in that I closed the bladder wound completely, first with a row of fine chromicised gut interrupted sutures, and then reinforced it with mattress sutures, the first layer extending to the mucous membrane, but not through it. The skin wound was partially closed, gauze drains being carried down to the bladder sutures properly to drain in case of bladder leakage. A No. 24 French catheter was inserted into the bladder through the urethra.

I believe many bladders we do not close, can be closed, provided the patient has a urethra that can stand continuous bladder drainage. My principal reason for closing the bladder was the fact of urinary fistula which might result, due to the residual urine from his prostatic obstruction. It might be said that his prostate should have been removed, but as he had had one fairly serious operation, and another very serious operation in less than two months, this did not seem advisable. His convalescence was uninterrupted, the continuous catheterization being kept up for ten days; his bladder wound healed perfectly and there was never any suprapubic leakage. His highest temperature was 101° F. and highest pulse 100.

At present he is in excellent condition, has five ounces residual urine, which is clear, although it contains microscopic pus, but no blood. He passes his urine once during the sleeping hours and every three or four hours during the day.

I have included in this series a case (Case VII) that was operated in for a tumor of the fundus of the bladder nine years ago. This growth was under the mucous membrane and attached to the fundus of the bladder to the right of the median line. It was pedunculated and encapsulated. Microscopic examination showed adenofibroma undergoing malignant change. This patient is well and in good health today. Doctor Welch, of Johns Hopkins, saw this tumor and thought it probably originated from the urachus.

I have recently seen a man fifty-five years of age with a carcinoma in the fundus and right lateral wall of the bladder. This man was in an advanced stage, metastasis having taken place in the prevesical space and extended down into the pelvis. So advanced was it that it was possible to palpate the tumor mass through the abdominal wall. As the patient was suffering so frightfully from tenesmus, a suprapubic operation was done for drainage.

Of my eight cases of cancer involving the fundus or lateral walls, three have been operated in by the method here described; the patients are all alive and well; one operated upon March 19, 1910, one November 11, 1910, and the last one March 18, 1912. As far as I have been able to ascertain, the method adopted in attacking these growths has not been tried before. This operation is applicable to the upper two-thirds of the bladder. It is not difficult to see, through the cystoscope, the dimpling of the bladder wall when pressed upon, and as soon as the threaded needle is passed into the bladder and traction is made on the suture, its position is readily noted. It has certainly made the operative treatment much more satisfactory than guessing at the point of location of the tumor, making the incision, having the bladder wall collapse, and finding the sutures are either too near or too far from the growth, and having to introduce the finger into the bladder to make out this line of incision and have the growth break up. I feel sure that the traumatism to the growth during its removal is one of the weakest features in the operative treatment of tumors of the bladder, and there is no question that by this method, unless there is a very contracted bladder, the operation is not difficult for one accustomed to using the cystoscope.

Of the eight patients reported whose growths were in the fundus of the lateral wall of the bladder, one, not operated on by this method, lived one year and died of metastasis to the throat; one not operated on on account of metastasis being present, died four days after I saw him; one operated on, the operation being abandoned on account of great extension outside of bladder wall, died of intestinal paralysis, ten days after operation; one operated on eight years ago, not by this method, is alive and well; an adenofibroma is undergoing malignant changes; three patients operated on by the method just described are living, with no sign of recurrence at this time; one three years seven months ago; one two years eleven months ago; and the third one year and seven months ago. The only hope for a patient suffering from vesical neoplasm is surgical intervention, and the success depends upon early diagnosis.

In conclusion, I feel that every patient who presents blood in the urine, unless there is a definite known cause for its appearance, should be examined at once and the source of the hemorrhage ascertained. If this rule is followd, I believe we shall not see so many hopeless cases of late vesical neoplasm.—New York Medical Journal.

204 The Farragut.

---

### SOME MANIFESTATIONS OF INFLUENZA IN YOUNG CHILDREN.

By L. Emmett Holt, M. D.

It is with some hesitation that one ventures to present a paper on so trite a subject as that of influenza, a term so widely used as a cloak for our ignorance of the explanation of obscure symptoms. I believe, however, that we are now in a position to clear up some of the haze which surrounds this subject and to chart some points at least in this indefinite sea of symptoms. That the term is constantly abused does not prove that there is no such thing as influenza. The same could formerly have been said of malaria. By influenza we mean an infection or inflammation due to Pfeiffer's bacillus, and only in this sense is the term used in this paper. The organism is one of those associated with inflammations of the respiratory tract and affects chiefly the lower tract, the trachea, bronchi and lungs, less frequently the upper respiratory tract, the naso-pharynx and ears. To apply the term influenza to an ordinary, severe head cold is a misnomer; in a very few cases is this organism found. As a rule, the B. influenza has not a high degree of virulence, but in exceptional instances this may be the case. At such times it may cause a general blood infection and not infrequently meningitis, more rarely joint inflammations.

The diagnosis of influenza is established only by finding the organism in the secretions; usually it must be the bronchial secretions. This renders its discovery difficult in young children. The bronchial secretion is not easy to secure. These patients will not cough and expectorate when desired, and sometimes several trials must be made before satisfactory material for cultures is obtained. The best way of accomplishing this in infants is to excite a cough by tickling the pharynx and to catch upon a pharyngeal swab any secretion brought up from the bronchi.

A diagnosis by the examination of smears is unreliable. Only cultures can be depended upon. It should be remembered that the B. influenzae grows only upon media containing haemoglobin (blood agar being that generally employed), so that cultures made upon ordinary diphtheria tubes are of no value. The difficulties of isolating the organism, even in cultures, are considerable and outside of a good hospital laboratory the work can hardly be done satisfactorily on any extensive scale. Hence it is not surprising that so few observations upon the definite diagnosis of influenza have been made.

At the Babies' Hospital during the past five years we have been greatly interested in the study of the bacteriology of respiratory infections. The accompanying tables gives the

Sputum Cultures for Five Years.

| | 1909-10 % | 1910-11 % | 1911-12 % | 1912-13 % | 1913-14 % |
|---|---|---|---|---|---|
| B. Influenzæ ..... | 32.5 | 32 | 33 | 28 | 42 |
| Pneumococcus .... | 62.5 | 66 | 81 | 80 | 87 |
| Streptococcus .... | 33.5 | 37 | 46 | 43 | 29 |
| Staphylococcus ... | 76.2 | 76 | 89 | 90 | 84 |

results obtained from 1,650 sputum cultures which have been made from 1,053 patients during the winter and spring seasons of this period. It shows the percentage of cases in which the different organisms, the pneumococcus, the streptococcus, the staphylococcus and B. influenzæ, have been present in the different seasons. While there is seen a general correspondence in the different years, it will be noted that there has been an unusual prevalence of influenza during the present season. This has been particularly true during the months of March and April. Our observations for the past five years indicate that influenza begins as the cold weather approaches, usually toward the end of October, but that it is not very frequent until after January. The spring months are usually the time when it is most often seen. Also that it disappears regularly with the advent of warm weather about the middle of May. Pneumococcus infections do not follow the same course, but are common during the year.

What does the presence of the B. influenzæ in the sputum signify? Some have said that it signified nothing; that the organism can hardly be regarded as pathogenic. Although this view was formerly held by many pathologists there are now few who maintain it. The occurrence of an acute purulent meningitis in which this is the only organism found and the production of a similar inflammation by its injection in animals with recovery of the organism, certainly establishes the fact of pathogenicity.

The B. influenzæ is only one of the common organisms associated with respiratory infections. It is seldom seen alone and it is therefore difficult to determine exactly which of the symptoms present may be fairly attributed to it. It is only by the study of a large number of cases in which the organism is found that this point can be settled.

Probably the most signficant manifestation of influenza is a peculiar range of temperature. The variations seen are most puzzling and frequently wrongly interpreted. They often give the physician the greatest concern, especially since they occur so frequently in the course of pneumania or otitis, they may lead to a suspicion that some serious complication, either medical or surgical, is present. The temperature is apt to be high, to fluctuate widely and irregularly without apparent cause. Its rise is sharp, but without chills; in its fall, which is quite as rapid, it frequently goes to subnormal. The want of correspondence between the general symptoms and the temperature is quite diagnostic. I know of no disease in which such high temperatures are seen with so few general symptoms as in influenza.

From our experience at the Babies' Hospital several definite clinical types stand out:

1. Pneumonias with unusual, often extraordinary, fluctuations of temperature or with a persistence of temperature after physical signs have disappeared.

2. Pneumonias running a protracted course, with slow resolution. Frequently there are recurring attacks.

3. Cases of otitis with very mild catarrhal symptoms, often only a moderate cough and a few coarse rales in the chest, but with a temperature quite out of proportion to the general or local symptoms.

4. Cases with very few or no catarrhal symptoms whatever, but with a very unusual temperature curve.

5. Unusual temperature curves accompanying tuberculosis and sometimes other diseases.

6. Cases resembling whooping cough, seen chiefly in older children, seldom in infants.

Let us consider this last-mentioned group first.

Pfeiffer's bacillus has many points of likeness to Bordet's bacillus. There are also clinical resemblances between whooping cough and influenza which I think have not been sufficiently appreciated. Influenza often persists for from six to eight weeks; it may be characterized by a paroxysmal cough, which is so like the paroxysms of pertussis that at times the two are indistinguishable. It is my own belief that most of the children who are reported to have recurrent attacks of whooping cough have in reality suffered from influenza.

There are some points of differential diagnosis which we may pause here to mention. The blood picture in whooping cough is quite different from that seen in influenza. Influenza without catarrhal symptoms is seldom seen, and then it has a relatively low leucocyte count, but with its usual bronchitis there is a leucocytosis of 18,000 to 30,000, the differential count showing sixty to seventy per cent of polymorphonuclears. In whooping cough there is a leucocytosis of about the same degree, but a very high lymphocyte percentage is regularly present.

Another point of resemblance is the contagious character of influenza and the fact that there may be in the same family two or three children with identical symptoms. A few years ago three children in a family all had at the same time typical whooping cough. During the following year all three developed a protracted paroxysmal cough, which, except for the previous year's experience, would unquestionably have been regarded as whooping cough. Sputum cultures from all three during the second season, however, showed the presence of the influenza organism. During the past few weeks a resident in a hospital where influenza was prevalent developed a paroxysmal cough of such severity that vomiting occurred two or three times a day. One physician who saw him made an unqualified diagnosis of whooping cough. His blood, however, showed 19,000 leucocytes with sixty-eight per cent of polymorphonuclears and his sputum contained great numbers of the influenza organisms, but not Bordet's bacillus, although careful examination for it was made. I think, therefore, that we should be extremely cautious in making the diagnosis of recurring attacks of whooping cough.

Since January, but especially during the months of March and April of this year, an unusual amount of influenza has been seen in the Babies' Hospital. In consequence of this, on April 19 and 20, sputum cultures were made from all the hospital inmates—doctors, nurses and patients.

Of ninety persons examined thirty-one showed the presence of influenza at this examination and three others had shown it previously, making a total of thirty-four infected persons, or thirty-eight per cent.

The most marked prevalence of influenza was in the wards in which the patients with bronchitis and pneumonia were received. In two such wards, containing twenty-one patients, ten had influenza. Of eight nurses in attendance in these wards five showed influenza. Six of the patients were suffering from bronchopneumonia, three others previously had slight temperatures not explained by the other symptoms present; a fourth case, one of tuberculosis meningitis, showed only a few influenza organisms. Two nurses had no symptoms suggesting influenza infection, but one, shortly after the culture was taken, developed a severe cold; the other three were suffering from catarrhal symptoms of various degrees of severity.

A nurse in charge of a special ward containing three children who were suffering from influenza showed the organism in her sputum. She had previously suffered from a mild cold.

Of the remaining twenty-five nurses in the hospital only four showed the B. influenzæ, and three of these had suffered from colds or a nasopharyngeal infection.

The resident physician had a severe paroxysmal cough and his sputum showed great numbers of influenza bacilli.

Of ten cases of pneumonia in the hospital at the time of the routine examination the sputum of eight showed the B. influenzæ. Of eight children admitted with pneumonia during the week following, five gave positive influenza cultures. Since the number of children admitted with influenza is so large the presumption is that the nurses contracted it from these children and then possibly spread the infection to others.

An unusual prevalence of influenza has been noted in some other institutions in the city where careful sputum cultures have been made. Dr. Swift, of the Rockefeller Institute Hospital, informs me that of thirteen pneumonia patients during the first three weeks of April eight showed the present of the B. influenzæ in the sputum cultures.

That the temperature curve and the course of pneumonia is influenced by the influenza complication, no one looking over these charts can for a moment question.

There are still many points to be settled regarding influenza infections. To call every case having an unusual temperature one of influenza is certainly a serious error; but that this infection causes very remarkable fluctuations of temperature in respiratory infections should certainly be recognized. Such temperature curves, I believe, are constantly misinterpreted.

Uncomplicated influenza has a good prognosis; but when influenza occurs as a complication of pneumonia it certainly increases the danger of that disease, particularly on account of the tendency to prolong its course and to increase the liability to recurrent attacks. One can never be quite sure when these patients are entirely well. The influenza organism we have found to persist for months in the sputum of children and during this time there was a constant tendency to recurrence of the catarrhal symptoms of greater or less severity.

Of treatment there is little to be said. Thus far it has seemed to me quite unsatisfactory. Its definite contagious character certainly increases the necessity for isolation. This is difficult to maintain, both in a hospital and a household, where nearly always there are several cases in a family. In hospital practice I am convinced that we have been negligent in not paying much greater attention to the separation of these cases from other inmates, for the added risks following infection in children are certainly considerable, not so much from the disease itself as from the complications with which it may be associated.

Cases of respiratory infections complicated by influenza are, in my experience, seldom benefited by cold air, although fresh air certainly is needed. They invariably improve when the warm days of spring come. If symptoms persist it is desirable to send children South to a warm climate. With others the plan of "watchful waiting" seems to give the most satisfactory results.

The accompanying charts illustrate the most frequent types of temperature seen in influenza cases. With one exception all of the patients have been observed during the past few weeks.

In none of these cases were any antipyretics given or any measures employed for an artificial reduction of the temperature. In all cases a careful examination was made of the urine, the ears, the digestive tract and the other organs to determine if possible whether there was any other factor present which might explain the temperature. The diagnosis of influenza was not admitted until all these had been excluded. In every case the diagnosis rested upon the finding of the influenza organism in numbers in the sputum cultures, and in most cases they were found repeatedly. The evidence seemed conclusive that influenza infection was a factor in the production of the clinical symptoms.

Case I was a well-nourished child of fourteen months, admitted with a lobar pneumonia at the left base; signs of resolution appeared shortly after the crisis, but the resolution was slow. The temperature continued some time after the lungs were clear. Influenza organisms were found in the sputum both during the height of the pneumonia and subsequently.

Case II was a well-nourished child of seven months, admitted on the twelfth day of his illness with a circumscribed consolidation of the right lower lobe. The only unusual feature was the irregular temperature curve. The lungs cleared up rapidly after the crisis. The influenza organisms were found in the sputum early in the disease, but not after the lungs had cleared.

Case III was a small but fairly nourished child of nine months, admitted in the third week of a pneumonia of the right apex; physical signs were typical. The temperature fell by crisis the day after admission. The early sputum cultures showed no influenza. The subsequent rise of temperature was accompanied by an increase in the catarrhal symptoms which followed a partial resolution, and at this time the sputum culture showed the influenza organism. In this case the child appeared to have contracted the infection in the hospital, as he occupied a bed next to a child who was found to have an influenza infection.

Case IV was one of tuberculosis peritonitis. The symptoms were typical, but no satisfactory explanation could be found for the unusual temperature curve. Frequent and repeated examination of the lungs and other organs showed no generalized tuberculosis, but, as influenza organs were present in the sputum in numbers, this was suspected. At autopsy there was no sign of a generalized tuberculosis, the lesions being essentially those of a tuberculosis peritonitis only.

Case V was an infant five months old, admitted with the diagnosis of tuberculosis meningitis. A clear fluid was drawn by lumbar puncture which contained a large number of cells, chiefly lymphocytes and tubercle bacilli. On the third day the temperature rose and continued high and fluctuating until death. The clinical symptoms resembled those of a cerebrospinal meningitis. A blood culture and a culture from the sputum both showed the B. influenzæ; the spinal fluid remained clear, but a smear contained a few organisms resembling influenza bacilli. The autopsy showed a tuberculosis meningitis at the base and a purulent meningitis at the convexity from which the influenza organism was obtained in pure culture. This case has been fully reported elsewhere.

Case VI is that of a child nineteen months old, who was admitted to the hospital as a case of mental deficiency. Neither cough nor fever had previously been present, nor were they present during the illness. On the day of admission the temperature was found to be 102° and quickly rose of to 106½°. There was a leucocytosis of 20,000 with sixty-six per cent of polymorphonuclears. The lumbar puncture was negative as well as the examination of the other organs. With this high temperature the child did not appear very ill, and when the temperature fell to or near normal the symptoms had quite disappeared. A second rise occurred a few days later with a repetition of the original symptoms and also without any special prostration. At this time also careful examination of lungs, urine, ears, were all negative.

Case VII was that of a delicate infant nine months old, admitted with a second attack of bronchitis, having been ill for a week. Otitis developed and double paracentesis was done on the third day, repeated again on the tenth. The left ear was again opened on the twelfth and twenty-fourth day. No explanation for the wide fluctuations in temperature could be discovered except the influenza, the sputum cultures showing the presence of the organisms. A small spot of pneumonia developed on the eighteenth day. Death occurred on the forty-fourth day. The autopsy showed no disease of the mastoid or of the sinus, but the cultures from the lungs showed the B. influenzæ and the pneumococcus.

DISCUSSION.

Dr. Linnaeus E. La Fetra, New York City: In the study of catarrhal infection of infants and young children there is great difficulty in determining the precise cause of infection, because several germs are usually present, and one must judge which is predominating. Moreover, few clinicians have the facilities for proper bacteriological study. Careful technique is particularly important with reference to the influenza bacillus, for it does not grow readily and seems easily to lose its vitality, especially if dried. Dr. Holt is particularly fortunate in having been able to study both clinically and bacteriologically such a large series of cases.

For a number of years it has seemed probable that a certain type of fever accompanying catarrhal infection in infants and children is due in many cases to the influenza bacillus. It is characterized by wide vacillations of temperature each day. I have seen it range from 106° to 94° in thirty-six hours. In many such instances the influenza bacillus has been found either alone or more commonly associated with others. It is fair to conclude that many other cases of analogous type in which bacteriological study was not made are probably due to the same cause. The general symptoms are fever and prostration, the fever being of the peculiar hectic type suggestive of deep suppuration. The prostration is quite marked, but the child's color does not suggest either suppuration or sepsis, and in the intervals when the temperature is low the child seems surprisingly well. The blood count shows an increased number of leucocytes with a high polynuclear count. With many infants this peculiar temperature and prostration are the only signs found throughout the course of the disease, there being no catarrhal symptoms whatever and no inflammation of the throat or of the ears. It would seem that the child was suffering simply from the general infection without localization. Such cases are very puzzling and are commonly attributed to a deep-seated pneumonia.

"Whooping bronchitis" is very interesting and it is certainly due in many cases to influenza; the catarrh of the lower trachea gives rise to the paroxysmal cough, often accompanied by a whoop.

Other types of the disease depend upon the addition of various local symptoms, such as gastro-intestinal catarrh, otitis media, in combination with catarrh of the upper respiratory tract. It is exceedingly important to remember this influenzal type of fever, since the wide range of temperature may suggest either serious mastoid involvement or the presence of sinus thrombosis. In this connection it is important to call attention to the fact that single blood counts, taken in connection with the high and widely fluctuating temperature, should not be relied upon for guidance as to the advisability of operation on the mastoid when there is influenzal otitis-media. The leucocyte count may be high, up to 15,000, and the polymonuclears may constitute seventy or eighty per cent without there being any involvement of the mastoid or of the sinus. Only if and when local signs in the ear canal and bone tenderness are present should the mastoid be operated upon. General symptoms of fever, prostration and high blood count are not a justification for operation.

Besides the catarrhal symptoms of the respiratory or gastro-intestinal tract a frequent manifestation is involvement of the lymph nodes of the neck. So long as the glands remain soft there is danger of another outbreak of fever or other complication, either in the lungs, ears or even in the meninges. One should be cautious about predicting a termination of the fever so long as the enlarged glands are present.

A rare and most unfortunate manifestation of influenza, either primary or as a complication, is meningitis. I have seen three such cases in the past five years and each presented features worthy of report. In the first of these cases the primary manifestation of trouble was a swelling of the small joints of the hands. This occurred in an infant eleven months old. Later other joints became involved and there was great prostration, with a vacillating temperature. There were very few evidences of meningitis, so that a lumbar puncture was not made. At the autopsy the joints and the brain showed a purulent exudate, which gave a few cultures of the influenza bacilli. Similar cases with joint involvement have been reported. The second case occurred in a boy of nine years and was seen by Dr. Holt also. This case began with a severe rhinitis, but soon presented an exact picture of cerebro-spinal meningitis of the epidemic type. Lumbar puncture withdrew a purulent fluid that gave a pure culture of influenza bacilli. Influenza organisms were recovered also from the secretions of the naso-pharynx. Several other members of the family had had severe attacks of the grippe. The third case occurred last fall in my service at Bellevue Hospital. The patient was a nursing baby five months old. The first manifestation was a convulsion and the symptoms were those of a mild case of epidemic cerebrospinal meningitis. The spinal fluid was turbid and so for two days after admission the ordinary anti-meningococcus serum was injected. There was, however, no improvement and the growth did not prove to be the meningococcus, but the influenza bacillus. Study of the spinal fluid at the Rockefeller Institute confirmed this finding and accordingly the anti-influenza serum was injected. This was not, however, until the tenth day after admission to the hospital and the quantity that could be injected within the next four days was very small on account of the local conditions of the spinal cord. The child failed rapidly and died on the fourteenth day after admission.

Dr. H. L. K. Shaw, Albany: Smears from the nasal and tracheal secretions in forty infants at St. Margaret's Hospital with catarrhal colds showed pneumococci. The reason why the influenza bacilli were not detected was due perhaps to the fact that no cultures were made. Recently I saw an infant with meningitis whose lumbar fluid showed a pure culture of influenza bacilli. An interesting feature of the case was that there were no previous symptoms of influenza in the baby or in any members of the family.

Dr. Herman Schwarz, New York City: Dr. Williams, of the research department for the New York health department, showed that in measles, scarlet fever, and other infections, the presence of influenza bacilli in secretions of the throat was more frequent in the winter time than during the summer.

It is interesting to note that the cultures from the middle ears in cases of influenza did not contain influenza bacilli. The cases complicated by glands did not show the presence of influenza bacilli in suppurative process of these structures.

Dr. DeWitt H. Sherman, Buffalo, asked Dr. Holt in reference to the prostration so commonly considered necessary as a sequellæ in influenza. He stated that in Buffalo this winter there has been a great deal of an infection associated with marked vacillation of temperature. When the temperature was high, whether morning or afternoon, the child felt ill, but when the temperature was low it felt almost perfectly well and wished to be up and about. These cases seemed to be influenza, but contrary to expectation made a prompt, complete recovery.—New York State Journal of Medicine.

### NO NEED TO FEAR MEAT.

### No Cattle With Foot-and-Mouth Disease Being Slaughtered in Federally Inspected Establishments. Thorough Cooking Will Render Uninspected Meat From Local Slaughter Houses Thoroughly Safe.

Washington, D. C., Nov. 18.—According to the specialists of the Department of Agriculture people even in States quarantined for the foot-and-mouth disease need have no fear of eating meat, provided they cook it thoroughly. The foot-and-mouth disease is not easily communicated to human beings through food, although milk from a diseased cow might transmit the disease to a human being. In the case of milk, however, pasteurization will render it entirely safe. Human beings who do get the disease commonly get it from direct contact with a sick animal. It is wisest, therefore, for people to keep away from all animals having the disease, unless they are properly provided with rubber gloves, coats and boots, and these are thoroughly disinfected after each visit to the animals.

In case of meat, as in the case of milk, it must be remembered that all herds which actually show the disease are quarantined, and neither milk nor meat from the sick animals can be sold. 60 per cent of the meat used in this country is produced in the nearly 900 Federally inspected slaughtering establishments located in 240 cities. In these establishments no animal is slaughtered until it has passed an ante-mortem inspection and also a most rigid post-mortem inspection by a veterinarian at time of slaughter. After slaughter its meat cannot leave the establishment until it has been carefully examined and stamped "U. S. Inspected and Passed." In all these establishments no animal showing any symptoms whatever of foot-and-mouth disease is allowed to go to slaughter, and no meat which, on post-mortem inspection, shows any suspicious symptoms of this complaint can be shipped out of the establishment. All meat suspected of coming from an animal suffering with this complaint is sent, under Government seal, to the tanks to be rendered into fertilizer. The Federal inspection stamp on meat, therefore, means that it is entirely safe.

The Federal Government, however, has no jurisdiction over local slaughter houses which do not ship meat outside of the State in which it is slaughtered. If, however, meat from such an animal did escape from one of these local slaughter houses, which are purely under State or munihipal control, all danger of its communicating the disease to human beings would be removed when it is thoroughly cooked and sterilized. Those who are located near an infected region and wish to be absolutely certain of the safety of their meat should cook it thoroughly.

The disease when contracted by adults is not at all a serious illness. It commonly takes the form of slight fever sores in the mouth and a slight eruption on the fingers. In the case of small or sickly children, it may take more serious form, especially if complicated by other illnesses.

---

### RECENT METHODS IN DERMATOLOGY.

By G. Norman Meachen, M. D., B. S., M. R. C. P., London and Edinburgh, Physician to the Hospital of the Skin, Blackfriars; Dermatologist to the Prince of Wales General Hospital, London, Eng.

If the advances made during the last few years in our knowledge of diseases of the skin, apart from those due to syphilis, have not been stratling, we may comfort ourselves with the fact that dermatology has at any rate, not lagged behind the other specialties of medicine. That link that binds dermatology to general medicine is largely a clinical one, but we have the great advantage both in diagnosis and treatment of being able to see what we are doing.

Excluding the Wassermann test and the luetin reaction of Noguchi, we have only the tuberculin test, whether injected into the patient or applied to the skin after the manner of Moro or Von Pirquet, that really deserves the title of specific. Considering the frequency with which a tuberculous focus is present somewhere in the body, a positive reaction to tuberculin should always, I think, be accepted with caution. Taken in conjunction with other evidence, it is useful in helping to confirm the tuberculous nature of a doubtful skin eruption. With regard to tuberculin-therapy, it may be said that if the practitioner wish for quick results he will be disappointed, but if he be prepared to peg away slowly, using, by preference, the bi-weekly gradually increasing dose, he may expect to see some improvement, if not an actual cure of a lupus. By the method of obtaining slight reactions only the chief objection to the use of tuberculin has been removed. At the recent meeting of the Dermatological Society of the British Medical Association, held at Aberdeen, Norman Walker related some successful cases of lupus treated with the local aplpication of a 5 per cent tuberculin ointment, and he said that he had not observed any marked local reaction.

The use of vaccines in general may justly be called specific, for what can be more so than the inoculation in a case, say, of sycosis, with a vaccine from a culture taken from the patient's own pus? In all staphyloccic infections of the skin, especially in furunculosis, vaccine-therapy generally yields good, and sometimes brilliant results. The method of Whitfield, described at the International Congress of Medicine in 1913, of giving 250 million staphylococci as an initial dose, increasing it rapidly, appears to me to be the method of choice. At the same meeting Gilchrist narrated his experiences with vaccines in skin diseases, and he finds that in the case of acne injections of a vaccine of 10-20 milion of acne bacilli as an initial dose, repeating the dose or slightly increasing it after the negative phase has passed, are the most efficacious. Some degree of local immunity seems to be produced by the application of ointments made up of various micro-organisms, such as staphylococcus albus ointment, 10 per cent in cold cream, in cases of seborrhœic eczema. Such methods seem reasonable and are worthy of trial.

THE ROLE OF PHYSICAL THERAPEUTICS.

The number of physical agents that have been employed of late years in the treatment of diseases of the skin is very numerous. One naturally thinks of X-rays first, and for the treatment of lupus vulgaris of the ulcerating type, rodent ulcer and skin cancers, and for producing epilation, for ringworm of the scalp and sycosis, this agent remains one of the best. Now that we have a better means of measuring the dose, injury to the skin is becoming progressively rarer, though there are cases still in which a delayed radio-dermatitis may appear, or where the hair fails to grow again after epilation.

In radium we have a remedy which far exceeds the X-ray in potency, and in the relief of itching and pain. Thanks to Wickham, Degrais, and other workers, sufferers from severe pruritus, chronic eczema, with lichenification, new growths of the skin, lupus, angeiomata, nævi, rodent ulcer, and other forms of malignant disease of the skin, have a reasonable chance of receiving benefit or lasting cure.

The Kromayer lamp, on account of the shortness of exposure required—about ten minutes—and the larger size of the area treated, bids fair to become a serious rival of, if not actually to supercede, the Finsen light. The ultra-violet rays from a mercury-vapor lamp, as originally employed by Kromayer and Nagelschmidt, have given excellent results in alopecia areata and in severe rosacea of a disfiguring type.

The good effects of hot air in dematological therapeutics are seen in the radiant-heat treatment of localized pruritis or chronic eczema. The "douching" of hot air by means of electricity is useful for limited areas of disease. More recently we have the method of through-and-through warming of the tissues, or diathermy, which, in the hands of Nagelschmidt, has given good results in cases of lupus, nævus, and rodent ulcer.

Many of these more advanced adaptations of light and heat rays demand the use of expensive apparatus, and are, therefore, only available for the favored few. If it be desired to obtain the benefits of local hyperæmia, one can employ Bier's suction cups at a small cost. These are small exhaust cups of glass, connected at one end with a rubber tubing. The size of the cup should correspond with that of the area to be treated, and only sufficient exhaustion should be produced so as to cause it to adhere. The cup is kept in situ for five minutes released, and re-applied after three minutes' interval for a further period of five minutes, for half to one hour, once or twice daily, according to the effect produced. Small patches of alopecia areata and most acne pustules may be satisfactorily treated by these suction-cups, though they will seldom take the place of local remedies entirely.

SOLID CARBON DIOXIDE.

The benefits of hyperæmia, combined with more or less destruction of tissue, can all be obtained by the use of what I may venture to call the most generally useful agent employed for this kind of work in the whole range of dermatological therapeutics. Introduced by Pusey, the solid crayon of frozen carbonic acid with a temperature of—79 degs. C., combines many of the properties of radium and X-rays without the expense of the one and the disadvantages of the other. Exactly how one prepares the solid snow is immaterial; I, for one, am fond of the Goosmann apparatus, where the receiver of the frozen gas also acts as an applicator. For nævi and moles it is unrivalled, while many other skin affections are benefitted by it, notably rodent ulcer, which it destroys in a very few applications, and also lupus erythematosus of the discoid variety. Warts and old-standing patches of lupus vulgaris require, of course, a longer application than that needed for superficial nævi. I do not think that solutions of the snow in acetone or ether possess any real advantage over the ordinary solid crayon. From the ease with which its application can be controlled it will be acknowledged that frozen carbon dioxide is superior, and, of course, far less costly, than liquid air. In the case of very young infants the exposure, and also the pressure, must necessarily be much less than in the case of adults.

### IONIC MEDICATION.

The researches of Leduc, Lewis Jones, and others, upon the electric penetration of ions into the epidermis has encouraged dematologists to employ the principles of electrolytic conduction of anions and kathions for the treatment of cutaneous disorders. The essentials of succes in ionisation are (1) to employ a weak solution of the substance chosen—seldom more than one to four per cent., (2) to protect the bare skin from the pole of introduction by several layers of moistened lint, (3) to see that no abrasion or irregularity of surface exist upon the area to be treated, (4) all oily and fatty matter must be removed from the skin by alcohol or ether before commencing the treatment. Zinc is a favorite metal for ionization, and a two per cent solution of the chloride or a four per cent solution of the sulphate is used for moistening the pad beneath the positive electrode, in this case made of zinc, placed over the part to be treated. A current of 2-3 milliamperes per square cm. is allowed to pass for ten to twenty minutes, the indifferent electrode being placed in a basin of salt water in which the patient immerses one hand. A siting is given once a fortnight, and in the case of a patch of lupus vulgaris, for which this method is eminently suitable, is repeated once a fortnight. In the case of rodent ulcer, the positive electrode may consist of a zinc needle which can be introduced beneath the skin into the diseased tissue.

Ionization with salts of mercury, copper or zinc, has been successfully employed in ringworm of the scalp, alopecia areata, warts, boils and sycosis. Ionisation with quinine has been employed with benefit in herpes zoster, while the salicylic ion has acted well in acne. Iodine ions have been used by Riddell, of Glasgow, for some time past in the treatment of ringworm.

### CHEMICAL AGENTS.

The setting free of nascent iodine locally by the action of peroxide of hydrogen upon a lupus lesion while the patient is taking iodide of sodium, after the manner of Pfannenstill, I have found of considerable value in dealing with nasal cases in which the mucous membrane was involved. At a recent meeting of the Dermatological Society of the Royal Society of Medicine a case was shown by Sibley in which lupus of the buccal mucous membrane had been successfully treated by insufflations of equal parts of iodide of potassium and chalk, the mouth being afterwards rinsed with chlorine water in lemonade. Axel Reyn lierates nascent iodine in a patient taking iodide of sodium by electrolysis of the lupoid lesion itself.

Some interesting observations have lately been made as to the power of certain aniline dyes to influence epithelial growth. One of these, scarlet red, has achieved considerale reputation as a cellproliferant, being employed as a 1-4 per cent ointment in cases of ulcus cruris. A modification of this, amidoazotoluol, or the diacetyl derivative of this known as pellidol, may be employed in weaker dilutions than scarlet red. Basic fuchsin has also been recommended by May and Heidingsfeld.

Allantoin, the active substance of the common comfrey root (Symphytum officinale) has been emplayed successfully by C. J. Macalister and others in chronic ulcers. It should not be used in foul or septic conditions, nor in too strong a solution.

Of late the remarkable researches of Strauss, of Barmen, into the germicidal powers of copper salts, especially in connections with tuberculosis, have led to the experimental application of copper chloride and tartrate, and, more recently still, the combination of copper with lecithin, known as "lecutyl," in cases of lupus. A little girl with severe lupus around the ankle has just been under my care in the hospital. I scraped her and afterwards applied copper lecithin locally. At the same time she took pills of the same substance internally. The time is young to report fully upon this method, but the results, so far, seem encouraging.

### AUTO-SERUM THERAPY.

Another direction in which a god deal of research has lately taken place is that of the injection of the patient's own blood serum in certain chronic dermatoses. It is now two years ago since Linser and Mayer were able to cure cases of urticaria, prurigo and herpes gestationis by this means. 10 to 20 c. c. of blood-serum are injected in these affections with success. Ravaut utilized the patient's own blood in similar cases. In certain toxic forms of skin eruption, such as occur in pregnancy, intramuscular injections of Ringer's solution have been practiced with success by Eichmann and others. Excellent results have been reported by Holobut and Lenartowicz in pemphigus by injecting the clear, sterilized contents of the bullæ themselves in 2-6 c. c. doses.

I have endeavored to indicate in the briefest fashion some of the principal directions in which progress is being made in dermatology. There yet remain a great many question to be investigated. The influence of the internal secretions, for instance, and the findings of chemical analysis of the urine, the fæces and the blood, the bacteriology of skin affections in general, and last, but not least, the discriminating use of histological methods, are all of the highest importance to the dermatologist who would study his specialty with keenness and interest. —Urologic and Cutaneous Review.

---

### DIFFICULTIES THAT THE ANESTHETIST HAS TO CONTEND AGAINST.

In the Australasian Medical Gazette of April 25, 1914, Hornabrook in discussing this topic says that the progress of anesthetic work has been greatly retarded by the freedom with which even difficult cases are handed over to men of not very profound experience, and the willingness of the profesion to shield itself, when a fatality does occur, under the plea that there was something abnormal about the patient or the anesthetic, instead of learning to combat that abnormality, and listening to men who have devoted a large proportion of their lives to endeavoring to improve the very unsatisfactory conditions at present existing. For some time past Hornabrook has been persistently advising anesthetists to keep to small doses of morphine and tropine as a preliminary to open ether work. This advice was not given for nothing, but was the result of close study of hundreds of cases extending over four years, and under various conditions, in the Melbourne Hospital, the Women's Hospital, the Eye and Ear Hospital, and in private work. The work was first started with 1-6 grain morphine and 1-120 grain atropine given from a quarter of an hour to one hour before operation. It was then found that this dose suited a large number of cases; there were others, especially anemic females, who showed signs of respiratory depression, not actually on the operating table, but from half an hour to one hour, and in some cases even longer, after the patient had been removed from the operating table in apparently excellent condition.

These cases caused him to reduce the dose to 1-8 grain morphine and 1-150 atropine, given from three-quarters of an hour to one hour before commencing the anesthetic during the sedative stage of the morphine, morphine being first a stlmulant, especially in small doses, secondly a sedative, and thirdly a respiratory depressant, so that by using the smaller dose there would be less likelihood of the morphine reaching the depressant stage. The atropine was also reduced, as it was found that in a few cases the y-120 grain caused the tongue to become too dry, and that the tongue then showed a tendency to swell and obstruct the airway. Moreover, with the mouth too dry, the anesthetic is not taken so easily.

Having used the 1-8 grain morphine and 1-150 grain atropine with very satisfactory results, he then endeavored to reduce it still further, and started 1-12 grain morphine and 1-200 grain atropine, and though this gave very satisfactory results in a number of cases, it was not so uniform as the 1-8 grain morphine and 1-150 grain atropine, and, moreover, there was a tendency for a little too much moisture in the air-passages, a result nearly as bad as a too dry tongue. So they returned to 1-8 grain morphine and 1-150 grain atropine, and this is used as a routine practice in the Women's Hospital, the Eye and Ear Hospital, and in the majority of cases in the Melbourne Hospital. The above is Hornabrook's maximum dose for all cases over twelve years of age, male or female. He finds this quite enough for any case if given at the right time, viz., three-quarters of an hour to one hour before operation. This preliminary of morphine and atropine makes the giving of the anesthetic far easier on the part of the patient, a most important point, and undoubtedly lessens the tendency to shock, even in the severest operations, and certainly reduces the tendency to acidosis and postoperative vomiting. Postoperative vomiting has today become almost a thing of the past, certainly as a persistent event. In using morphine and atropine the anesthetist must not use chloroform as an anesthetic per se; if he is so foolish as to do so, then he is certain to have a fatality sooner or later, not actually on the table, but very likely arising an hour or more after the operation has been completed, and the patient will die of respiratory failure. If he does not have a fatality, then he will have something very near to it, and considerable anxiety. Both morphine and atropine are respiratory depressants, and the man who persists in the use of this combination is doing a very serious wrong, and if a fatality occurs he renders himself liale to grave censure, even if some excuse is found in an abnormal condition of the patient.

Hornabrook had two fatal cases brought under his notice within the past twelve months, in which, in spite of the warning given above, the anesthetist had used 1-6 grain morphine and 1-120 grain atropine and chloroform as an anesthetic. Both cases were private ones, and females, and he has no hesitation in saying that their deaths were hastened by the method used, nor does he believe that either death would have occurred if the small dose of morphine and atropine has been employed, and open ether used as the anesthetic. Both fatalities occurred after the patient had een removed from teh operating table.

Recently Hornabrook has had two cases reported to him. In neither case did a fatality actually occur, but both caused considerable anxiety, and again instructions were not followed out, but 1-6 grain morphine used, and chloroform employed as the anesthetic. The mere fact that a man may have a large number of apparently succesful cases with 1-6 grain morphine and 1-120 grain atropine does not excuse him from using a smaller and safer dose, and, in Hornabrook's belief, quite as effective if given three-quarters of an hour to one hour efore operation.

In cases of severe shock it is a distinct gain to give the patient 1-8 grain morphine and 1-150 grain atropine about ten minutes before starting the operation. This is done to get the benefit of the stimulating stage of the morphine, and open ether should be employed as the anesthetic.

Hornabrook uses morphine and atropine for all his cases except short dental ones, postnasal and tonsillotomy operations. In the operation of enucleation of the tonsils, morphine and atropine given about an hour beforehand is of distinct advantage. The only exception to the use of morphine and atropine for operations of any duration is in those head cases in which there is any question of the respiratory center being out of gear, or the base of the brain involved; in these cases morphine must be avoided at all cost, though a small dose of atropine may be given with advantage.

The doses are as follows: Morphine 1-8 grain and atropine 1-150 grain for all patients over twelve years of age, and up to any age; morphine 1-12 grain and atropine 1-150 grain for eight to twelve years; morphine 1-20 grain and atropine 1-120 grain for 3½ to eight years; and it is advisable to use the tablets and not the made-up solution.

Hornabrooks has used morphine and atropine for a patient as young as fifteen months, but he does not advise it, as he has not yet had enough experience to speak with any certainty in cases below 3½ years. Of course, open ether is the anesthetic used, and not chloroform, even in children.

The anesthetist today takes other means to lessen the consumption of anesthetic by the patient. With the head and shoulders slightly raised at a natural angle from the commencement of induction, and, if possible, throughout the operation, he finds he requires less anesthetic and has, moreover, something to fall back upon should any trouble arise. Immediate lowering of the head will cause a rush of lood to the brain with stimulation of the cardiac and respiratory center, without the necesity of resorting to artificial respiration.

When a patient is once surgically anesthetized, the complete shutting off of all light from the eyes, by means of a thick towel or bandage, makes it very much easier for the anesthetist to maintain a successful anesthesia, and with a smalled consumption of anesthetic and less tendency to irritation of the air-passages, and also less tendency to postoperative vomiting. In the majority of instances the patient under operation lies with his face turned directly toward a bright light, and we all know how disturbing that is from our own experience when looking toward a bright light to feel the soothing effect, even when the lids are tightly closed. Personally, Hornabrook states, he never sees more of his patient's face than the lobule of one ear; attention is directed to this and respiration; the lobule of the ear shows trouble, whether cardiac or respiratory, at once, if any arise.

The whole object of the anesthetist today is to obtain a successful surgical anesthesia, using as little of his poisonous drugs as possible, and not seeing how much the patient will stand without killing him. By this means he helps considerably toward a successful convalescence, and should receive the thanks of the surgeon and patient; instead of which, if anything goes wrong, it is said to be due to faulty administration of the anesthetic.

There is one more word of warning, and that an important one. How many usrgeons ever think of informing the of asking if they may do so? And yet the surgeon who does anesthetist when they are going to use adrenalin, or even think not do this is acting wrongly. For all he knows the anesthetist may be using chloroform as his anesthetic, and chloroform with adrenalin is a very dangerous combination, possibly due to the weakened action of the heart from the chloroform, the contraction of the arterioles from the adrenalin, causing an obstacle that the heart in its embarrassed condition cannot overcome, and a fatality occurs. Hornabrook would especially warn against plugging the nostrils with adrenalin and cocaine, and then following with chloroform as a general anesthetic. Adrenalin can, however, be used with ether.

Again, do not use 1-4 grain or even 1-6 grain morphine suppository immediately on the termination of an operation if the patient has had any preliminary injection of morphine and atropine before operation, for the reason that you may find it is quite unnecessary, and a more important reason is that you are adding a respiratory depressant, which may lead to the necessity for artificial respiration after the patient has been removed to his room. It is far better and wiser to wait and give the suppository about five hours after the operation, when the danger is over. In a number of cases of hemorrhoids which have been under observation, it has been found, however, that the morphine suppository can very often be done away with.—Therapeutic Gazette.

---

### BIRTH RATES AND WAR.

In the year 1798, a country clergyman in England, by the name of Malthus, published an essay on the Principle of Population. This work immediately arrested the attention of the thinking world, and was later expanded into a volume of over five hundred pages, which was published in 1803. Although Malthus' work has been subjected to fierce criticism—most recently and most intelligently by Henry George, who, incidentally showed that the Irish famine was not due to overpopulation or the lack of abundant food in Ireland—the main contention of the theory that population tended to increase faster than the means of subsistence has not been and can not be controverted.

It is probably not because Malthus wrote his Principles of Population, but because of economic pressure among populations that never heard of Malthus, that we see the birth rate falling in almost all the nations of the allegedly civilized world. Not in all, but in almost all. Taking France as the most typical exemplar of a controlled birth rate, we find the population practically stationary—while the country, as a whole, is apparently self-sufficient, the people contented and eminently prosperous, emigration practically unknown, as France can furnish a good living to all Frenchmen who will work, and the state of arts and sciences are on the highest plane. There is no demand for conquest and expansion, as both statesmen and people recognize that France is sufficient for all those living within her European boundaries.

Taking, however, some other countries among the modern nations whose populations are increasing and we find, notwithstanding a reasonable degree of prosperity, a poticeable amount of restlessness and discontent. This seems to be fostered by the statesman, who, while the deprecate any „moral restraint" of Malthus which might curb the excessive birth rate, cause apprehension by pointing out the inevitable results of overpopulation. In line with this confused view of a question of fundamental importance, which was in the realm of theory a hundred years ago, but has now become the practice of intelligent and conscientious peoples, has arisen a school of popular writers of more or less authority according to their rank in official or academic life, who teach that it is the duty of women to bring forth even though and for the purpose that the country may be overpopulated, and this surplus population shall be the cause of and be used in war and conquest.

Some of these lucubrations cite the Old Testament as a divine authority for overpopulation, and all seem to regard Malthus' "moral restrain" as potential murder. As it is an ancient law that circumstances alter cases, it is only fair to point out that the circumstances of the ancient Israelites in Asia in no way resemble those of any modern States, and if modern man is to be bound by the injunctions delivered by

Jehovah to the Jews, modern life would have to revert to that of tent dwellers and nomadic shepherds, and we would be subject to laws of apparently equal authority on other subjects which we have long since disregarded with great benefit to ourselves.

The second question is a more delicate subject, for it invariably connects itself with Malthus' alternatives to moral restraint. The Reverend Fellow of Jesus College, Cambridge, says:

"On examining these obstacles to the increase of population which are classed under the heads of preventive and positive checks, it will appear that they are all resolvable into moral restraint, vice and misery. Of the preventive checks, the restraint from marriage which is not followed by irregular gratifications may properly be termed moral restraint. Promiscuous intercourse, unnatural passions, violations of the marriage ed, and improper acts to conceal the consequences of irregular connection, are preventive checks that clearly come under the head of vice. Of the positive checks, those which appear to arise unavoidably from the laws of nature, may be called exclusively misery, and those which we obviously bring upon ourselves, such as wars, excesses and many others which it would be in our power to avoid, are of a mixed nature. They are brought upon us by vice and their consequences are misery."

A flat contradiction by the political economy of the early nineteenth century of some of the neo-sixteenth century doctrines exploited in these early years of the twentieth century. Even granting that man is not yet sufficiently spiritual to be trusted with the commission of Malthus' first "obstacle to the increase of population"—and possibly this is the case—it would seem from what we now know about war (and on this side of the water it is only the man with imagination who is able faintly to visualize the reality) that Malthus' second alternative, villainous as its category is, is preferable to the third, which includes war. The latter now and always has included almost all the specifications of the former and it fits perfectly that supplication to the deity which admits with shame "there is no health in us."

If war is a necessary consequence of overpopulation, it is time then for man to see to it that overpopulation does not occur again. He has overcome the force of gravity in a myriad ways; he has overcome the darkness of night--electricity does his bidding in the remotest corners of the earth—he sails upon the sea and through the air, he has conquered almost all the forces of nature, but to what end if he does not conquer himself?

Fortunately, even in the most prolific countries, birth rates are beginning to fall, and presently a low birth rate will be one of the marks of culture in a nation as it now is in classes and families.—Lancet Clinic.

